# The Airway Microbiota in Cystic Fibrosis: A Complex Fungal and Bacterial Community—Implications for Therapeutic Management

**DOI:** 10.1371/journal.pone.0036313

**Published:** 2012-04-27

**Authors:** Laurence Delhaes, Sébastien Monchy, Emilie Fréalle, Christine Hubans, Julia Salleron, Sylvie Leroy, Anne Prevotat, Frédérick Wallet, Benoit Wallaert, Eduardo Dei-Cas, Telesphore Sime-Ngando, Magali Chabé, Eric Viscogliosi

**Affiliations:** 1 Center for Infection and Immunity of Lille (CIIL), Institut Pasteur de Lille, Biology and Diversity of Emerging Eukaryotic Pathogens (BDEEP), BP 245, Lille, France; 2 INSERM U1019, Lille, France; 3 UMR CNRS 8402, Lille, France; 4 Department of Parasitology-Mycology, Faculty of Pharmacy, University Lille Nord de France, EA4547, Lille, France; 5 Department of Microbiology, Lille Hospital, Faculty of Medicine, Lille, France; 6 LMGE, Laboratoire Microorganismes: Génome et Environnement, UMR CNRS 6023, Clermont Université, Blaise Pascal, BP 80026, Aubière, France; 7 Université Lille Nord de France, Université du Littoral Côte d'Opale, ULCO, Laboratoire d'Océanologie et de Géoscience (LOG), UMR CNRS 8187, Wimereux, France; 8 Genoscreen, Institut Pasteur of Lille, Lille, France; 9 Department of Biostatistics, Lille Hospital, Faculty of Medicine, Lille, France; 10 Department of Pneumology and Immuno-Allergology, CRCM adulte, Calmette Hospital, Lille, France; University of Edinburgh, United Kingdom

## Abstract

**Background:**

Given the polymicrobial nature of pulmonary infections in patients with cystic fibrosis (CF), it is essential to enhance our knowledge on the composition of the microbial community to improve patient management. In this study, we developed a pyrosequencing approach to extensively explore the diversity and dynamics of fungal and prokaryotic populations in CF lower airways.

**Methodology and Principal Findings:**

Fungi and bacteria diversity in eight sputum samples collected from four adult CF patients was investigated using conventional microbiological culturing and high-throughput pyrosequencing approach targeting the ITS2 locus and the 16S rDNA gene. The unveiled microbial community structure was compared to the clinical profile of the CF patients. Pyrosequencing confirmed recently reported bacterial diversity and observed complex fungal communities, in which more than 60% of the species or genera were not detected by cultures. Strikingly, the diversity and species richness of fungal and bacterial communities was significantly lower in patients with decreased lung function and poor clinical status. Values of Chao1 richness estimator were statistically correlated with values of the Shwachman-Kulczycki score, body mass index, forced vital capacity, and forced expiratory volume in 1 s (*p* = 0.046, 0.047, 0.004, and 0.001, respectively for fungal Chao1 indices, and *p* = 0.010, 0.047, 0.002, and 0.0003, respectively for bacterial Chao1 values). Phylogenetic analysis showed high molecular diversities at the sub-species level for the main fungal and bacterial taxa identified in the present study. Anaerobes were isolated with *Pseudomonas aeruginosa*, which was more likely to be observed in association with *Candida albicans* than with *Aspergillus fumigatus*.

**Conclusions:**

In light of the recent concept of CF lung microbiota, we viewed the microbial community as a unique pathogenic entity. We thus interpreted our results to highlight the potential interactions between microorganisms and the role of fungi in the context of improving survival in CF.

## Introduction

The human respiratory tract represents the major portal of entry for numerous microorganisms, primarily those occurring as airborne particles such as viral and bacterial entities, or fungal spores. Microorganism characteristics coupled with the local host immune response will determine whether they will be cleared or adhere and colonize the airways leading to acute or chronic pulmonary disease.

In cystic fibrosis (CF), mutations in the cystic fibrosis transmembrane conductance regulator (CFTR) gene result in defective mucociliary clearance and, as a consequence, lead to the production of thick and sticky bronchial mucus, which facilitates the entrapment of airborne viruses, bacteria and fungal spores and provides a suitable environment for the growth of these microorganisms. In addition to bacteria, which are well known to cause recurrent exacerbations of CF-associated pulmonary disease and often determine the vital prognosis of patients [1], many fungi also colonize the respiratory tract of CF patients [2]–[5], although their involvement in respiratory infections remains controversial and largely unsolved [6], [7]. As an alternative to conventional methods (direct examination and microbiological cultures), new molecular techniques such as pyrosequencing, have been developed to offer a more complete view of the microbiota. In human samples, these molecular methods can distinguish microorganisms difficult to identify and/or those that are refractory to culture (such as *Pneumocystis jirovecii*, *Scedosporium apiospermum*, atypical mycobacteria, etc.), as well as new or as yet unknown pathogens [8]–[10]. The metagenomic approach has been recently used for the identification of human bacterial populations in the gut as well as in the mouth saliva and skin of patients [11]–[15]. In addition, metagenomic studies have been successfully used for providing an overview of community composition with semi-quantitative information [13], [14], [16]–[19]. Some studies have been published on the human respiratory tract, but only few have specifically focused on microbial diversity in CF [16]–[18], [20]–[25].

In the present study, we applied a molecular approach by pyrosequencing variable regions of bacterial 16S rDNA and fungal ITS2 genes in sputum samples from CF patients. Our aims were to explore the fungal and bacterial assemblages in CF patients to achieve a better understanding of species/taxon diversity and population dynamics of the microbiota, and their relevance for the clinical course of pulmonary disease in CF.

## Results

### Samples and patients

We prospectively collected eight sputum samples from four CF adult patients (median age of 29.5 years; Q1, 24.5; Q3, 34) who were all part of a long-term follow-up program at Lille's Adult CF center. Two temporal sputum samples were collected from each clinically stable patient with a sampling interval of 1 year; for Patient 4 the sampling interval was only three months (Tables 1 and 2). Three out of the four patients were homozygous or heterozygous for the ΔF508 mutation.

**Table 1:** Clinical data and treatment from CF patients included in the study.

Sample Identification	Clinical data from CF patients					Standard Spirometry			Treatment					
Patient- Date (mm/yyyy)	Age (year)	Sex	Known CFTR mutation	S-K score^a^	BMI^b^ (kg/m^2^)	SaO2(%)	FVC^c^ (%)	FEV^d^ (%)	ATB^e^ courses during the previous year		Corticosteroids		Azithromycin	ATF^f^ (days)
									IV	**po**	**Inhaled**	**Systemic**		
1-01/2008 Patient1-sample1	29	F	ΔF508 ΔF508	55	19.9	95	64	42	2	1	0	0	1	ITC^g^ (183)
1-01/2009 Patient 1-sample 2	30			50	18.6	96	61	40	2	1	0	0	1	ITC (183)
2-03/2008 Patient 2-sample1	38	M	ΔF508R347H	90	20.8	98	118	114	0	0	1	0	0	0
2-03/2009 Patient 2-sample 2	39			ND	21.5	96	114	112	0	0	1	0	0	0
3-09/2007 Patient 3-sample 1	29	M	ΔF508ΔF508	90	25.5	96	109	68	0	1	1	0	0	0
3-09/2008 Patient 3-sample 2	30			85	24.2	96	86	58	0	1	1	0	0	0
4-08/2008 Patient 4-sample 1	19	F	UNK^h^ UNK	50	15.2	97	35	28	7	1	1	1	1	0
4-10/2008 Patient 4-sample 2	20			45	15.6	91	34	48	1	1	1	0	1	0

a S-K score, Shwachman-Kulczycki Score;

b BMI, body mass index;

c FVC, forced vital capacity;

d FEV1, forced expiratory volume;

e ATB, antibiotic drug;

f ATF, antifungal drug;

g ITC, itraconazole;

h UNK, unknown CFTR mutations associated with an abnormally high sweat chloride test (110 mmol/L).

**Table 2:** Microbiological data from CF patients included in the study.

Sample Identification	Conventional analysis of sputum				
	Bacteriological culture	Mycological culture		Molecular analysis	
Patient- sample	Bacteria	DE^a^	Fungi	Nested PCR^b^	rt-PCR^c^
					
Patient 1-sample 1	*Pseudomonas aeruginosa* (mucoid texture) *Alkaligenes xylosoxidans*	0	*Candida albicans Geotrichum sp*	−	−
Patient 1-sample 2	*P. aeruginosa* (mucoid texture)	0	*C. albicans*	+	−
Patient 2-sample 1	ND^d^	0	*C. albicans*	+	+
Patient 2-sample 2	*Haemophilus influenzae*	0	*Aspergillus fumigatus C. albicans*	+	+
Patient 3-sample 1	*Staphylococcus aureus* (sensitive to meticillin)	0	*A. fumigatus Aspergillus flavus*	−	+
Patient 3-sample 2	*S. aureus* (sensitive to meticillin)	PH,H^e^	*A. fumigatus C. albicans*	−	+
Patient 4-sample 1	*P. aeruginosa* (mucoid texture)	0	*C. albicans*	+	−
Patient 4-sample 2	*P. aeruginosa* (non-mucoid texture) *P. aeruginosa* (mucoid texture)	H	*C. albicans A. fumigatus*	−	+

a DE, direct examination;

b Nested PCR was used to identify *Pneumocystis jirovecii* colonization [26];

c rt-PCR, real-time polymerase chain reaction assay to detect *Aspergillus fumigatus* [27];

d ND, not done;

e PH, Pseudo-hyphae and H, hyphae.

Results of the Shwachman-Kulczycki score (S-K score), body mass index (BMI), forced vital capacity (% of predicted FVC), and forced expiratory volume in 1 s (% of predicted FEV1) expressed as medians (Q1, Q3) were 55.0 (50.0; 90.0), 20.4 (17.1; 22.85), 75.0 (48.0; 111.5), and 53.0 (41.0; 90.0), respectively. None of the CF patients had pancreatic alterations. All patients had bacteria present in their sputa, as determined by culturing, but none were known to be chronically infected with *Staphylococcus aureus* or *Burkholderia cepacia* complex. Using conventional methods, we identified mucoid and non-mucoid *Pseudomonas aeruginosa*, meticillin-sensitive *Staphylococcus aureus*, *Haemophilus influenzae*, and *Alcaligenes xylosoxidans* (Table 2). No oropharyngeal flora was detected, and no mycobacteria were isolated. Two patients (Patients 1 and 4) were colonized by *P. aeruginosa* and treated with azithromycin, as recommended for chronic *P. aeruginosa* infections. Both patients had severe airway disease as assessed by the S-K score (S-K score ≤50%), BMI (under 16 for Patient 4) and standard spirometry (FEV1<50% of predicted FEV1) (Table 1). Patients 2, 3 and 4 were treated with inhaled corticosteroids, and Patient 4 received systemic corticoids (Table 1). Only Patient 1 received long-term itraconazole treatment (600 mg per day for 6 months) for allergic bronchopulmonary aspergillosis (ABPA).

Regarding fungi, *Candida albicans* and *Geotrichum* sp., and two filamentous species, *Aspergillus fumigatus* and *Aspergillus flavus*, were isolated from sample cultures. *Aspergillus nidulans*, *Aspergillus terreus*, *S. apiospermum*, *Scedosporium prolificans*, or *Exophiala dermatitidis* were not isolated. In addition, *P. jirovecii* colonization was retrospectively diagnosed in three out of four patients. Both sputum samples of Patient 2, as well as one of Patient 1 (sample 2) and Patient 4 (sample 1) were nested PCR-positive for *P. jirovecii* (Table 2) [26]. *Aspergillus* DNA was detected using an ultrasensitive real-time PCR assay [27] in five of the eight sputum samples (Table 2).

### Overall richness and diversity of microbial community evaluated from pyrosequences

**Figure 1:**
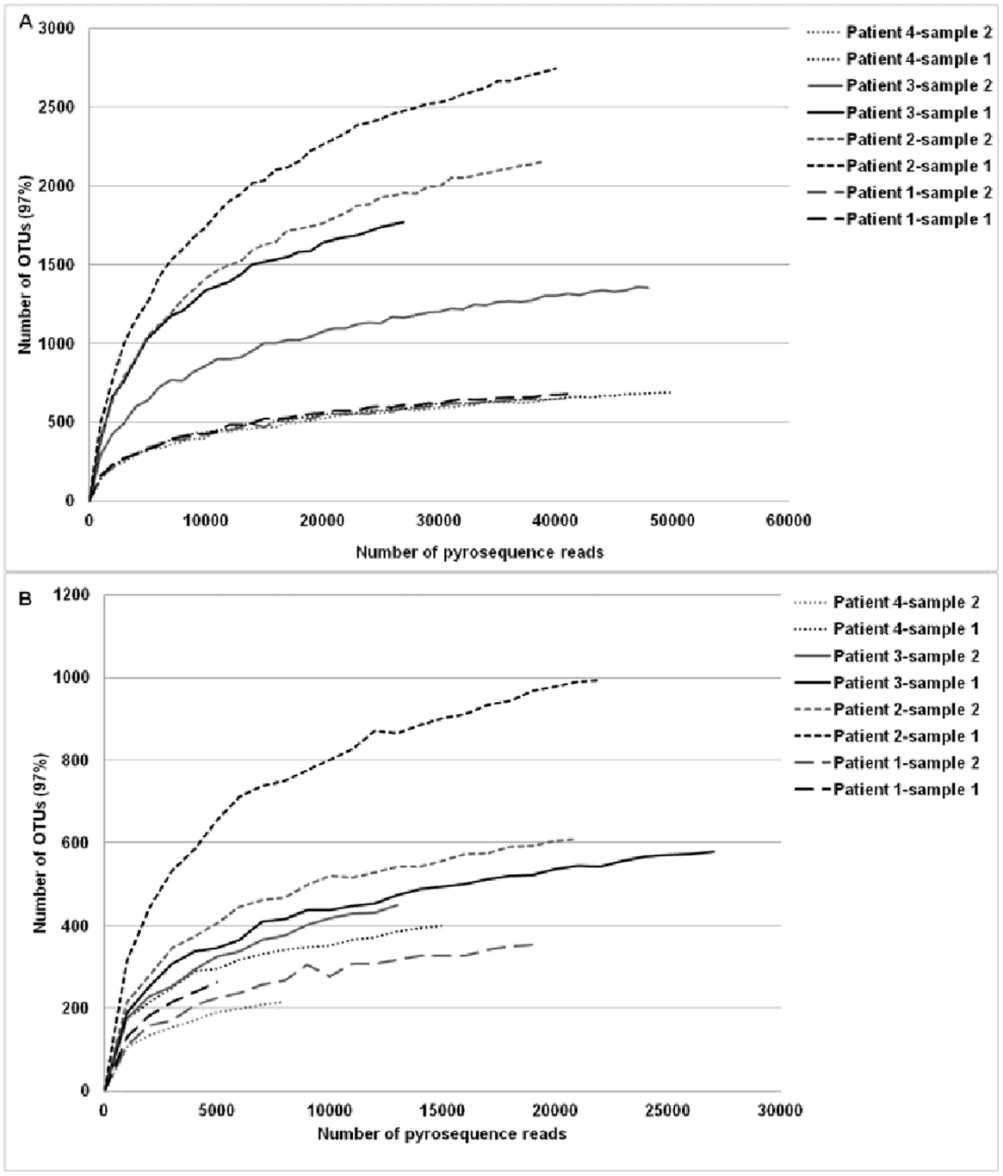
Rarefaction curves. These curves are representing the numbers of OTUs with respect to the number of pyrosequence reads obtained from each patient at different sampling times and using the two set of primers targeting prokaryotic 16S rDNA (A) and fungal ITS2 (B) loci.

We obtained a total of 326,277 sequences from samples 1 and 2 of Patients 1, 2, 3 and 4 using primers for the prokaryote 16S rDNA gene, a result in agreement with recent published data [17] (Figure 1A). Using the fungus-specific ITS2 primers, we obtained a total of 133,317 sequences from these samples (Figure 1B). Once primer, tag and key fragments were removed, 93% and 85% of the sequences had lengths greater than 450–500 bp and 300–450 bp for the 16S rDNA and ITS2 loci, respectively.

The pyrosequences that presented similarities with sequences available in databases but that could not be classified to at least the level of kingdom using BLASTN and MEGAN software were designated as “not assigned” and excluded from subsequent diversity analyses. For each sputum sample, these sequences represented less than 5% of the 16S rDNA or ITS2 sequences included in analyses, except for Patient 1-sample 2 and Patient 2-sample 2, which showed 9.7% and 8.4% of non-assigned 16S rDNA and ITS2 sequences, and 29.4% of non-assigned ITS2 sequences, respectively. Pyrosequences without any similarity with sequences available in databases were designated as “no hits”, and may represent species not yet represented in databases. Unsurprisingly, there were more ‘no hits’ for ITS2 pyrosequences than in 16S rDNA pryosequences (Tables 3 and 4, Figures S1, S2, S3, S4), due to the massive amount of data available in the Silva SSU rDNA database compared to the ITS2dbScreen database created expressly for the present analysis (see Materials and Methods). Un-represented organisms in sequence databases have already been described as a limitation in the ability to placing reads in the phylogeny [28].

**Table 3:** Number of 16S-pyrosequencing reads assigned to each taxonomic group of Bacteria.

	Sequences (reads)^a^ per patient and per sample							
Identification	Patient 1		Patient 2		Patient 3		Patient 4	
	Sample1	Sample2	Sample1	Sample2	Sample1	Sample2	Sample1	Sample2
**ACTINOBACTERIA**								
Actinomycetales	0	7	17	10	0	0	0	0
Actinomyces	6	11	47	44	32	0	0	0
Rothia	0	0	458	158	15	0	0	0
Atopobium	0	0	9	0	0	0	0	0
**BACTEROIDETES**								
Bacteroidales	0	0	10	7	0	0	0	0
Porphyromonas	0	0	145	0	0	29	0	0
Prevotellaceae	0	0	15	15	27	14	0	0
Prevotella	98	8	784	2098	620	11	10	0
**FIRMICUTES**	0	0	38	8	20	0	0	0
Bacilli	0	0	32	0	17	0	0	0
Bacillales	0	0	5	0	0	0	0	0
Gemella	0	0	113	22	79	0	10	0
Lactobacillales	0	0	12	16	9	0	0	0
Enterococcus	0	0	40	10	30	0	0	0
Streptococcus	5	7	255	931	446	5	6	0
Clostridia	0	0	0	0	0	0	0	0
Clostridiales	0	0	11	39	35	0	0	0
Mogibacterium	0	0	6	0	0	0	0	0
Eubacterium	0	0	0	0	11	0	0	0
Catonella	0	0	0	0	18	0	0	0
Veillonellaceae	0	0	0	8	0	0	0	0
Megasphaera	0	0	7	33	0	0	0	0
Veillonella	8	6	63	236	104	6	0	0
**FUSOBACTERIA**								
Fusobacterium	0	0	46	19	6	5	0	0
Leptotrichia	0	0	0	5	5	0	0	0
**PROTEOBACTERIA**	47	41	30	19	6	51	50	67
Betaproteobacteria	6	7	0	0	0	0	0	0
Alcaligenaceae	6	0	0	0	0	0	0	0
Neisseriaceae	0	0	0	0	14	0	0	0
Neisseria	0	0	35	15	0	5	0	16
Campylobacter	0	0	6	7	118	0	0	0
Gammaproteobacteria	1349	4622	68	9	0	5370	1303	1666
Pasteurellaceae	0	0	255	83	0	11	0	0
Haemophilus	0	0	124	5476	0	5	5	0
Moraxella	0	0	74	0	0	0	0	0
Pseudomonas	5851	230	0	0	0	833	6744	8298
Stenotrophomonas	0	0	0	0	0	5	0	0

a Once a read was assigned to the highest taxonomical level according to the criteria defined in material and method section, it was not added up in the next taxonomic level.

**Table 4:** Number of ITS-pyrosequencing reads assigned to each taxonomic group of Fungi.

	Sequences (reads)^a^ per patient and per sample							
Identification	Patient 1		Patient 2		Patient 3		Patient 4	
	Sample1	Sample 2	Sample1	Sample2	Sample1	Sample2	Sample1	Sample2
**DIKARYA**	9	7	36	14	10	2	52	18
**ASCOMYCOTA**	0	14	31	26	18	50	5	12
Saccharomyceta	6	257	129	199	7	4	38	107
Pezizomycotina	0	0	1	0	0	2	0	1
Leotiomyceta	1	1	1722	216	89	188	0	104
Dothideomycetes	0	1	10	7	0	0	0	0
Cryptococcus	0	0	0	0	0	0	0	145
Didymella	0	117	0	0	0	0	0	0
Phaeosphaeria	0	0	7	0	0	0	0	0
Eurotiomycetes	0	0	18	0	0	0	0	0
Eurotiomycetidae	0	0	4	0	3	7	0	0
Eurotiales	0	0	0	1	3	5	0	1
Trichocomaceae	0	0	120	462	1108	2661	0	129
Eurotium	0	0	13	0	0	0	0	0
Mitosporic Trichocomaceae	0	0	9	5	6	2	0	4
Aspergillus	0	0	403	0	13	8	0	15
Penicillium	0	0	25	306	0	0	0	0
Neosartorya	0	0	0	557	1887	5179	0	239
Sordariomyceta	0	0	2	0	0	0	0	0
Helotiales	0	0	9	0	0	0	0	0
Chalara	0	0	17	0	0	0	0	0
Sclerotiniaceae	0	0	0	69	0	0	0	0
Sordariomycetes	8	0	0	7	0	0	0	0
Hypocreales	4	0	0	0	0	0	0	0
Nectria	16	0	0	0	0	0	0	0
Xylariales	0	0	0	0	1	0	0	0
Physalospora	0	0	0	0	5	12	0	0
Saccharomycetes	0	0	1	0	0	0	3330	0
Saccharomycetales	9	60	92	60	198	0	808	116
Dipodascaceae	11	0	0	10	0	0	0	0
Clavispora	0	0	139	0	0	0	0	0
Candida	202	8688	5126	7167	6078	0	1173	6916
Saccharomycetaceae	12	0	4	0	0	0	398	0
Kluyveromyces	483	0	0	0	0	0	0	0
Saccharomyces	0	0	0	0	0	0	8	0
Torulaspora	0	0	20	0	0	0	0	0
**BASIDIOMYCOTA**	1	0	104	29	0	0	2	74
Agaricomycotina	0	0	13	1	0	0	2	5
Agaricomycetes	0	0	477	16	0	0	58	20
Hyphodontia	0	0	0	0	0	0	488	0
Coriolaceae	0	0	2	0	0	0	0	0
Piptoporus	0	0	103	30	0	0	0	0
Phlebiopsis	0	0	0	0	0	0	0	42
Russulales	0	0	1	0	0	0	0	0
Peniophora	0	0	204	0	0	0	0	0
Stereum	0	0	33	0	0	0	0	0
Agaricomycetidae	0	0	2	0	0	0	0	0
Agaricales	0	0	2	0	0	0	0	0
Physalacriaceae	0	0	1	0	0	0	0	0
Strobilurus	0	0	6	0	0	0	0	0
Tremellomycetes	0	0	91	0	0	0	0	0
Dioszegia	0	0	129	0	0	0	0	0
Sporobolomyces	0	0	7	0	0	0	0	0
Microbotryomycetes	0	0	1	2	0	0	4	0
Sporidiobolales	0	0	11	5	0	0	52	0
Sporobolomyces	0	0	0	0	0	0	8	0
Ustilaginomycotina	9	0	8	14	0	10	0	3
Entylomataceae	0	0	2	0	0	0	0	0
Entyloma	0	0	73	0	0	0	0	0
Malassezia	473	0	201	302	0	338	0	75
Microstromatales	0	0	0	0	0	0	0	9
Quambalaria	0	0	0	0	0	0	0	14

a Once a read was assigned to the highest taxonomical level according to the criteria defined in material and method section, it was not added up in the next taxonomic level.

For all patients and samples except one (i.e. Patient 1-sample 1 for the ITS2 locus), the rarefaction curves for the number of OTUs per pyrosequence reads reached a plateau, indicating that almost all OTUs present in each sample were detected. The apparent observed diversity was higher for the prokaryote 16S rDNA locus (Figure 1A) in comparison to the fungus-specific ITS2 locus (Figure 1B).

**Figure 2:**
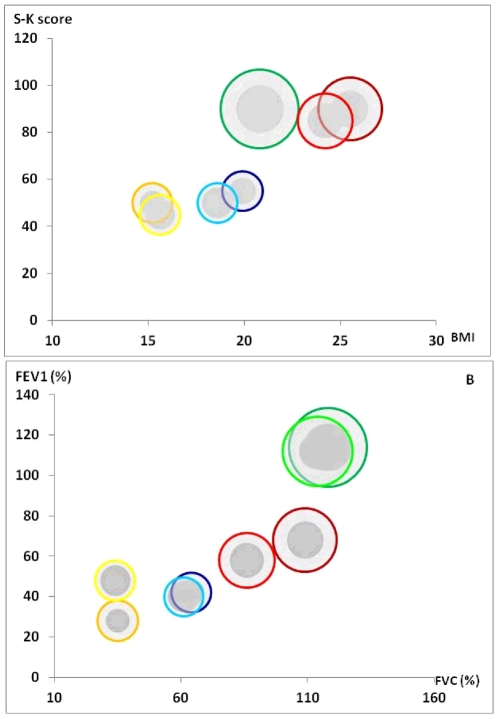
Relation between species richness and clinical status (A) or lung function (B). Total richness of prokaryotic and fungal communities from each patient-sample was expressed using the Chao1 richness estimator; each spot size is proportional to the corresponding Chao1 value. The clinical status is expressed as S-K score and BMI in Figure 2A, while lung function is expressed as FEV1 and FVC values in Figure 2B. Given to the absence of S-K score value from Patient 2-sample 2 (Table 1), this spot is missing in Figure 2A. Bacterial and fungal Chao1 values corresponding to Patient 1, Patient 2, Patient 3, and Patient 4 are represented in blue-, green-, red- and yellow-edged spots, respectively. Dark and light colour intensity is corresponding to the first and second sampling dates of each patient, respectively. Dark grey and light grey are corresponding to fungal and bacterial Chao1 richness values, respectively.

Calculated to analyze microbial diversity, Chao1 richness estimator values corroborated rarefaction curves, confirming high bacterial diversity (Figure 2A). Bacterial diversity was higher in samples from Patients 2 and 3 than in samples from Patients 1 and 4. Fungal diversity showed a similar pattern (Figure 2B).

### Comparison of the culture and pyrosequencing results

Our results confirmed the new genera recently identified in CF patients [8], [16], [17], [20], [23], [24], [29]–[31], with *Gemella* sp. being found in sputum samples of 3 out of 4 patients (Table 3, Figures S1A–S4A). The most represented genera identified in the present study were *Pseudomonas*, *Streptococcus*, *Haemophilus*, and anaerobes, in agreement with published data [1], [16], [17], [20], [23], [24], [30], [31]. Bacteria belonging to *Pseudomonas*, *Streptococcus*, *Prevotella*, *Fusobacterium*, *Haemophilus*, *Veillonella*, and *Porphyromonas* genera were isolated as recently reported in either sputum or BAL samples from CF patients (Table 3) [16], [18], [20], [21], [23], [30].

High fungal diversity was also observed in samples, with more than 60% of the fungal species or genera obtained in pyrosequencing not identified by mycological cultures (Tables 1, 2 and 3, Figures S1B–S4B). Among the 24 species or genera of micromycetes identified by pyrosequencing, only four were also isolated in culture. Using the metagenomic approach, we identified additional species, especially within the genera *Candida* and *Aspergillus*, which are microorganisms known to be involved in pulmonary diseases or infectious diseases [3], [4], [32]–[45]. *Geotrichum* sp., which represents an important pathogenic genus with teleomorphs assigned to *Dipodascus* [35], was identified to the family level using the pyrosequencing method (Table 4, Figure S1B), due to the stringent parameters chosen to assign ITS2 sequences. Phylogenetically distinct from the *A. fumigatus* cluster, non-*fumigatus Aspergillus* isolates were molecularly identified in Patient 2-sample 1 (Figure S5), in agreement with RT-PCR results (Table 2), which detects the mitochondrial DNA of *A. fumigatus* as well as other species such as *A. flavus* [27]. *Aspergillus lentulus*, which represents a species difficult to differentiate from *A. fumigatus* solely based on phenotype criteria but has decreased susceptibility to azoles [45]–[47], was isolated from Patient 3-sample 2 (Figure S3B). The major expected advance from the high-throughput sequencing method was its ability to identify difficult-to-culture micromycetes, such as *P. jirovecii* or *Malassezia* sp. Although nested-PCR targeting *P. jirovecii* was positive in 4 sputum samples (Table 2), high-throughput sequencing did not identify this fungus, probably because there is only one copy of the ITS2 locus in the *Pneumocystis* genome [48]. *Malassezia restricta* was identified in all patients (Table 4, Figures S1B–S4B), and *Malassezia globosa* and *Malassezia sympodialis* were molecularly identified in Patient 2 samples (Figure S2B). These results are consistent with the lipophilic nature that characterizes these yeasts and prevents their growth in standard culture media because they require an exogenous source of fatty acids [35]. *Malassezia* spp are frequently found in the skin of warm-blooded vertebrates, and they are currently recognized as emerging infectious pathogens [4], [35]. Recently, *Malassezia* has been identified in sputum samples from CF patients [25].

Since results from the conventional and high-throughput sequencing techniques concurred, the pyrosequencing method was used to identify dominant taxa, estimate their diversity, and analyze their temporal distribution, based on data obtained from both bacterial and fungal primers.

### Fungal diversity and associated patterns of bacterial flora

The relative amounts of each species were estimated from the number of assigned pyrosequences, and were represented by pie charts whose diameters are proportional to the number of assigned sequences (Figures S1, S2, S3, S4). According to recent publications [19], [22], the number of pyrosequences obtained corresponds to the number of genome copies present in the sputum sample. The median (Q1, Q3) number of microorganism genera per sputum sample was 3.5 (3; 7.5) micromycetes and 6.5 (5; 13.5) bacteria; these results were comparable to those of previous studies [1], [8], [9], [29], [31]. We observed bacterial diversity similar to that recently reported in CF patients using molecular methods [1], [8], [17], [20], [23], [24], [29]–[31], with anaerobic bacteria representing a large proportion of the detected species (ranging from 2% to 50% of total pyrosequences for Patient 1-sample 1 and Patient 3-sample 2, respectively). For the kingdom Fungi, the 133,317 pyrosequences corresponded to 30 species or genera, including 24 micromycetes and 6 basidiomycetous macroscopic fungi. Among them, filamentous fungi belonging to the genera *Aspergillus* (in particular *Aspergillus fumigatus*), and *Penicillium* have already been described as pathogens in CF patients [2]–[5], [35], [45]. *Candida albicans* and species from the *Candida parapsilosis* complex have been recently recognized as medically important organisms colonizing CF patients [2], [4], [40], [42], [43]. Although their clinical relevance is still matter of debate, long-term persistence of *Candida* strains have been described in CF respiratory tracts [4], [40], [42], [43], [49]. *Clavispora* is a yeast genus that includes *Clavispora lusitaniae* (teleomorph of *Candida lusitaniae*); this ascomycete has already been isolated from sputa [36], [40].

A significant proportion of other species were either fungi reported in asthma (*Didymella exitialis*, *Penicillium camemberti*), allergy diseases (*Aspergillus penicilloides* and *Eurotium halophilicum*) [32], [33], [37], [39], [50], or infectious diseases (*Kluyveromyces lactis*, *Malassezia* sp., non-*neoformans* Cryptococci, *Chalara* sp.) [34]–[36], [41]. The other species or genera represented environmental taxa, either described as wood-inhabiting fungi common in temperate regions of the Northern Hemisphere, such as cereal pathogens associated with air pollution or food preparation processes [51]–[53]. In addition, macromycetes living on the wood of tree species common in Europe including in northern France, were identified in sputum samples from Patients 1, 2, and 4 (Figures S1B, S2B, S4B), probably corresponding to the signature of the outdoor environment that the patients are exposed to [54]–[56].

A growing number of studies has revealed that bacterial [1], [8], [17], [20], [23], [24], [29], [30], [57], [58] and fungal [9], [25] community compositions vary greatly among patients. Diversity at sub-species levels has also been described in CF, mainly for bacteria such as *P. aeruginosa* [17], [57], [58], and to a lesser degree for fungi [9], [25] or viruses [18]. Therefore, the microbial community was currently considered to be a unique pathogenic entity with potential interactions between microorganisms [17], [59]–[61]. From the perspective of this microbiota concept, we phylogenetically analyzed the diversity of the main fungi and bacteria identified by pyrosequencing, considered the taxon composition of each sample with potential interactions between fungi and bacteria, and investigated its clinical significance.

### Population dynamics of the microbial communities in CF airways and clinical relevance

Although we observed lower diversity in CF airways than in other communities such as human skin, gut, or water microbiomes [12], [14], [19], reduced diversity and richness of fungal and bacterial communities were associated with poor clinical status, as evaluated from the S-K score and BMI values, and decreased lung function, as measured by FEV1 and FVC values, in CF (Figure 2). Chao1 values were statistically correlated with values of the S-K score, BMI, FVC, and FEV1 (*p* = 0.046, 0.047, 0.004, and 0.001, respectively, for Chao1 indices of fungal species, and *p* = 0.010, 0.047, 0.002, and 0.0003, respectively, for Chao1 values of bacterial species). Moreover, fewer fungus species were detected in sputum samples with lower FEV1 values; the correlation trended toward significance (*p* = 0.062). In parallel, a significant correlation between the number of bacteria species detected per sputum sample and values of S-K scores, BMI, FVC and FEV1 was observed (*p* = 0.0005, 0.03, 0.0003, 0.016 respectively) in agreement with published data [23], [58].

As previously observed [1], [30], anaerobes were significantly isolated in association with *Pseudomonas*, when comparing the relative amount of reads in each sample (p = 0.0003). Using a phylogenetic method, most *Pseudomonas* pyrosequences proved to be highly similar and clustered with sequences of *P. aeruginosa* strains isolated from CF patients or endotracheal tube biofilms (Figure S6) [62]. They also exhibited high infraspecific diversity, in agreement with previous results [57], [58]. The next most common bacterial genus was *Streptococcus*, of which the *Streptococcus milleri* group (SMG) has been isolated in CF [1], [20], [30], [63], linked to pulmonary exacerbations [1], [63], and demonstrated to produce quorum-sensing signal molecules [63]. SMG-related *Streptococcus* were identified in Patient 2-sample 2 and Patient 3-sample 2 (Figure S7). These phylogenetically identified SMG members (their sequences clustered with the SMG sequences of *Streptococcus anginosus*, *S. intermedius*, and *S. constellatus* in figure S7, using Neighbor-joining approach) were not numerically dominant compared to other clades, in agreement with the clinically reported absence of pulmonary exacerbation. Phylogenetic analysis of pyrosequences corresponding to the genera *Haemophilus* and *Malassezia* did not provide any new information compared to the pyrosequencing analysis using BLASTN and MEGAN software.

Using the same phylogenetic method, we observed diversity among genotypes of *C. albicans*, *C. parapsilosis* and *A. fumigatus*, with the same genotypes shared between patients, and/or genotypes that persisted over time within patients (Figures S5, S8, S9), in agreement with published data [40], [64]. *Candida albicans* and *C. parapsilosis* represented typical dominant yeasts isolated from CF sputa [2], [4], [5], [40], [65] for which we observed diversity similar to that already reported (mainly a single predominant *C. albicans* genotype) [40]. Regarding the aspergilli, samples were mainly composed of *A. fumigatus* as shown in the phylogenetic analysis (Figure S5), except for Patient 2-sample 1 in which the *Aspergillus* genus showed a high diversity, including non-fumigatus *Aspergillus* (Figure S2B, and sequences in dark green in Figure S5). Among *A. fumigatus* pyrosequences of Patient 3, one genotype was predominant in both samples of the patient, in agreement with previous studies that have demonstrated the emergence of a single genotype from a multiple-genotype population when chronic infection has been established [64], [66], [67].

Several recent taxonomic studies have identified cryptic species within key clinical morpho-species of both yeast and molds, including the *C. parapsilosis* complex, the *A. fumigatus* species complex and the *S. apiospermum* complex, which are particularly involved in CF lung colonization [46], [47], [68]–[70]. Here, we were able to differentiate *C. metapsilosis* genotypes from *C. parapsilosis* genotypes (Figure S7), as well as *A. lentulus* from *A. fumigatus* (Figure S5). This may have therapeutic implications given the different antifungal susceptibility profiles of these species [40], [45], [71].

The relative amounts (expressed as percentage of reads in each sample) of *C. albicans* or *A. fumigatus* were not statistically correlated with any bacterial taxon, neither anaerobic bacteria, nor *Pseudomonas*, nor *Streptococcus*. Nevertheless *C. albicans* was frequently associated with *P. aeruginosa* (80% of cases), which may be related to its recently proposed core status [21] and the bidirectional signalling pathway observed [for review60], [72]–[75]. Patient 3-sample 2 had a high number of *A. fumigatus* pyrosequences (23.6%) and this was associated with a predominance of *Streptococcus* (44.4%), which is a genus known to produce quorum-sensing molecules and to induce interactions between microorganisms, particularly among SMG members isolated from CF patients [63]. Regarding the temporal changes in the microbiota in each patient, we observed similar patterns, namely a disappearance of or major decrease in some bacterial genera recently described as members of the “core” pulmonary microbiome [76] and known to be a part of the oral bacterial community coupled with the emergence of more pathogenic bacteria (loss of *Prevotella* in Patients 1, 2, 3, and 4, *Gemella* in Patients 2, 3, and 4, *Veillonella* in Patient 3 associated with the emergence of *P. aeruginosa* in Patients 1 and 3, and of *H. influenzae* in Patient 2). Similarly, *C. albicans*, *A. fumigatus* or *A. lentulus* were detected in the second samples of Patient 1 and Patients 2, 3, and 4, respectively, while fungal species or genera known to be poorly pathogenic disappeared.

On the whole, our study highlights the correlation between richness and diversity of fungal and bacterial microbiota (Figure 2). We therefore suggest that “colonization resistance” occurs in CF lower airways, similar to what has been proposed to explain the exclusion of pathogenic species from the gut and the mouth by the presence of a specific microbiota [59], [77]–[79]. This phenomenon may be due to a range of factors and microbe-microbe interactions, including the presence of “synergens” described as enhancing the pathogenicity of the whole microbiota [78], [79], that will be discussed below.

## Discussion

Given the recent evidence that fungi may be of clinical relevance in the decline of CF lung function, associated with co-colonization of fungi and bacteria [5], [22], [49], [80], we coupled fungal analysis to the characterization of bacterial flora in sputum samples from CF adults using the pyrosequencing technique. We acknowledge that the present CF cohort is small but comparable to sample size recently published (from 4 to 14 sputum samples [17], [23], [76]), lacks a specific control group — which is difficult to choose [7], e.g. there can be extensive overlap of bacterial membership between the pulmonary microbiome of healthy subjects and patients with or without COPD [76] —, and probably is not completely representative of the full spectrum of CF pulmonary pathology. However, this pyrosequencing-based study of fungal and bacterial communities in the human airway confirmed the recently reported bacterial diversity (including anaerobes) in CF patients [8], [17], [20], [23], [24] as well as in COPD patients using BAL [76], and revealed complex fungal biota in sputum samples, with a majority of the fungal species or genera obtained by pyrosequencing not identified in cultures, most of them known to be pathogens. Using phylogenetic tools, we also found infraspecific diversity in *C. albicans*, *C. parapsilosis* and *A. fumigatus* similar to previous published data [40], [42], [64], [66], [67]. In parallel, cryptic and new unculturable (or difficult to grow *in vitro*) species have also been identified, most of them described as human pathogens. In agreement with a recent oligonucleotide array analysis [9], we showed that fungal microbiota colonizing the lower airways of CF patients is more diverse and complex than previously estimated with culture methods. Therefore, culture methods are probably inadequate for assessing CF respiratory fungal microbiota, although culture methods can be improved with increased standardization [3], [81] and are still required to determine drug susceptibility. Moreover, we have evidence that poor clinical status is associated with lower taxon diversity and richness in fungal and bacterial communities (decrease in S-K scores, BMI, FVC, and FEV1 values significantly associated with low Chao1 indices).

Our findings add support to (i) the pathogenicity of species derived from the oral cavity and usually considered as clinically insignificant such as anaerobes and SMG members, even if their role in infection and inflammation needs to be further elucidated [1], [8], [17], [20], [23], [29], [31], [63], [79], and (ii) the complex interaction between typical pathogens and microbiota, such as the association between *P. aeruginosa* and anaerobes [20], [30], [58], [59]. Since *C. albicans* and *C. parapsilosis* can also be part of oral flora, these yeasts can migrate from the oral environment, colonize and persist within the lower airways of CF patients [40], as proposed for bacteria [23]. Although the implication of *C. albicans* in the decline of CF lung function has been recently suggested [49], the clinical relevance of yeasts is still matter of debate, and remains to be confirmed. Given the airborne transmission of molds such as *A. fumigatus*, opportunistic molds represent the most common agents of fungal colonization and/or infection of the CF airways. Among them, *A. fumigatus* has been reported more and more frequently since the 2000s [3]–[5], [9], and is associated with clinical significance in CF [80] and modification in the population of genotypes during chronic colonization [64], [66], [67]. Fungal colonization (especially repeated or chronic colonization) may have a substantial impact on the development of CF pulmonary disease [43], [49], [80], but more studies are required to determine this fungal risk, especially in light of the concomitant bacterial biota.

Given the relationship between decreased microbiota diversity and poor clinical status, we hypothesize that the composition of the microbial community in CF airways is the result of dynamics that take into account the different microorganisms present as an entity with interactions at the intra-species level as well as at the inter-species level. This is somewhat similar to the constitution of oral microbial consortia for which the potential for infection or co-infection is realized when potential pathogens find suitable community partners and local conditions (host response, adhesion receptors, biofilm formation) [59]. It is well known that the heterogeneity of mucus composition in CF provides suitable conditions for chronic infection by a wide range of microorganisms. In particular, recent data indicate that reduced oxygen tension in CF lung promotes the growth of *P. aeruginosa* [82], [83], as well as other anaerobic bacteria [1], [30]. *Candida albicans* can also grow under anaerobic conditions, showing mating type modifications that may promote yeast development [84], [85].

In addition to local physical conditions, both bacteria and fungi possess the ability to form biofilm consortia [63], [82], [83], [86]–[88]. In this context, direct and indirect microbe-microbe interactions have been well documented, particularly those involving the major prokaryotic CF pathogen: *P. aeruginosa* (for review, see [89]). *Pseudomonas aeruginosa* can produce substances that modulate growth of other microorganisms, in particular fungi. *Pseudomonas aeruginosa* and *C. albicans* can coexist, or have an antagonistic influence as recently proposed between *P. aeruginosa* and *A. fumigatus* [60], [72], [90], [91]. Moreover, *C albicans* produces farnesol, which in addition to its quorum-sensing function regulating yeast morphogenesis and its ability to modify *P. aeruginosa* growth, also reduces competition from other fungi such as *A. fumigatus* [92]. Because a large proportion of bacteria have been shown to synergistically affect CF disease outcome by modifying the expression of virulence genes [79], it may not be surprising to find evidence of such synergistic interactions within the fungus community.

Thus, analyzing microbial diversity in polymicrobial samples such as CF sputa is the type of study for which metagenomic methods have been recently proposed [16], [18], [21], [78]. Our results, along with others [16]–[18], [24], [76], demonstrate the utility of high-throughput sequencing in identifying microorganisms to investigate the microbiome associated with chronic pulmonary diseases, such as CF or COPD. These results now need to be confirmed by further pyrosequencing studies, especially in large multicenter studies that will lead to a better understanding of the dynamics of such CF microbiota.

In the near future, microbiota complexity should be taken into account to analyze host-microbe interactions, which are bi-directional and probably not limited to the direct contact lung area (as proposed in ref. [61]). The analysis of CF pulmonary disease and its management should be reassessed in light of these interactions. This concept of CF lung microbiota has emerged recently from the scientific community working on the microbiology of the CF respiratory tract [61], [78], and entails coupling environmental microbiological approaches with community ecology analyses (i.e. analyzing species richness and relative species abundance in terms of either spatial or temporal distribution and dividing species into core and satellite groups, at an ecologically relevant spatial scale) [23], [31], [61], [76]. Furthermore, these molecular results should be combined with biological models, such as biofilm models or in vivo planktonic cultures as recently proposed, in order to elucidate the possible interaction between bacteria and fungi detected here [59], [63], [82], [83], [86]–[93].

Few culture-independent strategies have been developed to evaluate bacterial [1], [8], [16], [17], [20], [23], [24], [29], [31], fungal [9], and viral [18] diversities in sputum samples from CF patients. Thus, new high-throughput sequencing approaches offer more exhaustive coverage of the sequences present in PCR products, in particular when the new generations of automatic sequencers, such as the GS FLX Titanium System, are used. Compared to terminal restriction fragment length polymorphism (T-RFLP) analysis, high-throughput sequencing methods more accurately identify pathogens, because they are based on sequences instead of amplicon sizes that can be shared between two or more species. For example, *S. sanguinis*, *S. parasanguinis* and *S. salivarius* all generate a 576 bp T-RFLP fragment [1].

Nevertheless, these molecular strategies can have some confounding factors. One important drawback due to the basic PCR approach is the incapacity to reflect the viability of the microorganisms detected by DNA amplification, unless samples are pre-treated (with, for example, propidium monoazide, [61]). Furthermore, DNA extraction from clinical samples is the first crucial step in ensuring faithful molecular detection. Non-homogenous lysis of bacterial and fungal cells, which are known to require strong lysis in order to extract DNA, may introduce biases as in any method based on DNA amplification [94], [95]. In addition to DNA extraction efficiency that can vary between microorganisms, the choice of the PCR protocol, from primer design to the number of PCR cycles, can affect the results. In contrast to specific PCR targeting a specific pathogen, high-throughput methods as well as cloning/sequencing techniques, are based on amplification with primers targeting conservative regions of microorganism DNA. These techniques can thus identify any microorganism that is reasonably abundant within the sample without the need for prior prediction of which species may be present. This universal-primer approach leads to the preferential amplification of the most prevalent flora. This bias may explain the negative pyrosequencing results for *P. jirovecii*, which may be present in small numbers since only nested-PCR was positive (not detected upon direct examination). The clonal Sanger-sequencing approach would be more suitable than pyrosequencing methods for identifying microorganisms in relatively low abundance [8]. Improvements in amplicon length with the next generation of sequencers will determine the capacity to analyze amplicon diversity and to assign amplicons to species instead of genera. Additionally, the prominent advantage of pyrosequencing is its automation, which leads to increased standardization, from DNA extraction to sequencing analysis, allowing multicenter studies to be carried out at without compromising reproducibility.

### Conclusion

The aim of microbiological diagnosis from CF patients is to provide data with which clinicians can make rational and effective therapeutic decisions. Given the currently acknowledged polymicrobial nature of CF sputa [1], [8], [9], [17], [29], [31], better knowledge of sputum microbiota would represent a major advance in our understanding of the disease. In light of this concept of CF lung microbiota [61], [78], [96], high-throughput sequencing, due to its potential for massive direct sequencing after a single run of DNA amplification and automation, appears to be the most promising approach. The present study should stimulate a debate over the best way to set up new studies with the aim of combing (i) new technology (deep-sequencing), (ii) ecological tools (to analyze dynamics, diversity and relative species abundance, as species distribution is ecologically important in terms of community interactions [31], [78]), and (iii) clinically relevant information (e.g. pulmonary exacerbation in which SMG bacteria have been implicated when chronic colonization by *P. aeruginosa* develops a loss of virulence [1]) as well as the impact of therapeutics (long-term antibiotics cause a decline in bacterial diversity and inadvertently allow *P. aeruginosa* to flourish [58]; little is known about the impact of azole on fungal biota in CF).

Clearly, further metagenomic research, for which a scientific framework is needed as are well-designed translational studies, is now warranted to enhance knowledge of the process that drives the progression of CF respiratory disease. A comprehensive view of bacterial plus fungal microbiota present in CF lower airways has the potential to dramatically improve survival in CF patients. Moreover, it will pave the way for developing personalized drug therapy strategies based on the manipulation of complex microflora (i.e. controlling growth of less desirable microorganisms or controlling biofilm-associated infections as recently proposed [59], [89]).

## Materials and Methods

### Sample collection and DNA extraction

Patients were eligible if they could be classified as clinically stable (i.e., being followed-up during their annual check-up without exacerbation status). All volunteers with CF were required to have a well-documented diagnosis, with either the two mutations identified in the CFTR gene or an abnormally high sweat chloride test (Table 1). The four CF individuals selected for the study consisted of two males and two females, with an age range of 19 to 39 years. All clinical, therapeutic, radiological, and biological data were collected by clinical staff at the time of the visit (Tables 1 and 2). Human sputum samples (two samples collected for each patient at two visits) were collected by expectoration into a sterile cup after a water rinse to prevent excessive salivary contamination, [17], [23], [31]. Sputa were homogenized for 30 min at 37°C with Digest-EUR® (Eurobio, France) in 1∶1 (v∶v) ratio (final volume of approximately 10 ml), and mycological cultures were performed after direct examination, as previously described [4]. Briefly, 20 µl aliquots of the digested sample were inoculated onto three growth media: CandiSelectTM4 (Bio-Rad; incubation at 37°C for 3 weeks), Sabouraud glucose peptone agar with 0.5 g/L amikacin (incubation at 25°C for 3 weeks), and 1∶2 diluted Sabouraud glucose agar with 0.5 g/L amikacin (incubation at 25°C for 3 weeks). All sputa were inoculated in parallel onto five agar plates including chocolate Poly ViteX agar, Columbia colistin-nalidixic acid agar, Bromo Cresol Purple agar, blood agar (all purchased from bioMérieux, France) and incubated at 37°C for 48 h) and Cepacia agar (purchased from AES Laboratory, France), and incubated at 30°C for 5 days). Colonies growing on these media were identified using conventional methods or spectrometry. Then, 200 µL of each digested sample were frozen at −20°C until use. Samples were first ground in liquid nitrogen with a mortar and pestle. DNA was then extracted using the High Pure PCR Template Preparation kit (Roche Applied Science, Germany) according to manufacturer's protocol, except for the proteinase K digestion step, which was performed for 1 h at 70°C rather than 10 min. Total DNA concentrations ranged from 50 to 75 ng/µL, using NanoDrop® ND-1000 spectrophotometer. A nested PCR targeting *Pneumocystis jirovecii*, a difficult-to-culture micromycete, and a real-time PCR targeting *Aspergillus fumigatus*, were retrospectively done as described previously [26], [27]. No significant PCR inhibitions were observed when DNA samples were diluted in 1/10.

### Ethics Statement

Sputa from four CF patients who volunteered for the study were collected at the Lille Adult CF center, in accordance with the ethical guidelines of Lille University Hospital. This study was part of the “MucoFong” protocol and was approved by the Institutional Human Care and Use Committee of the Lille University Hospital (Comité de Protection des Personnes Nord Ouest IV - reference Number CPP 06/84; assurance number: SHAM 127795). Written informed consents were provided by study participants.

### Pyrosequencing analysis

Two sets of primers were used to amplify the 16S rDNA and ITS2 loci from prokaryotes and fungi, respectively. The first set of primers, 3271-16S-F (TACGGRAGGCAGCAG) and 3271-16S-R (GGACTACCAGGGTATCTAAT), was designed to amplify a 465 bp region containing the complete V3 domain of all prokaryotic 16S rDNA genes [97]. The second set, composed of primers 3271-ITS2F (CARCAAYGGATCTCTTGG) and 3271-ITS2R (GATATGCTTAAGTTCAGCGGGT) was designed to amplify a 340–360 bp fragment of the ITS2 region from all major phyla of fungi, according to the use for reconstructing phylogenies at a higher taxonomical level of this region [98]. A 10 bp tag specific to each of the eight samples, a 4 bp TCAG key, and a 21 bp adapter for the GS FLX system, were added to the sequences of both primers sets. PCRs were carried out using standard conditions for Taq DNA polymerase with 10 ng of DNA as template. After the denaturation step at 95°C for 5 min, 35 cycles of amplification were performed with a GeneAmp PCR System cycler (Applied Biosystems) as follows: 30 s at 95°C, 30 s at 50°C and 1 min at 72°C. Each DNA sample was analyzed in duplicate. The Genoscreen company (Pasteur Institute of Lille, France) carried out the pyrosequencing. The library and the 454 GS FLX Titanium (Roche) pyrosequencing runs were prepared according to manufacturer's recommendations. We obtained 326,277 and 133,317 sequences with the first (16S prokaryotes) and second (ITS2 fungi) set of primers, respectively. The sequences or reads were classified according to the presence of the tag corresponding to each of the eight samples of interest. Primers, tag and key fragments were not included in sequence analysis.

For identification, the 16S rDNA gene sequences were compared to the Silva SSU rRNA database (http://www.arb-silva.de/) release 102 (updated on February 15, 2010) comprising 1,246,462 SSU rRNA sequences using BLASTN software [99]. For ITS2 sequence identification, we constructed a fungal ITS2 database, based on the following steps: (i) a search through the complete nucleotide database of GenBank for potential ITS2 sequences, (ii) selection of ITS2 sequences that included the sequences of the primers designed in the present study, and (iii) inclusion of human genome sequences that were 500 bp long with at least one of the two primers to filter sequences belonging to host human cells (indicated as “Homo sapiens” in the final taxonomic assignment of the pyrosequencing ITS2 reads). This ITS2 database, named ITS2dbScreen, is available on request *via* the web site of the Genoscreen company (www.genoscreen.fr).

BLAST results (with a 10^−5^ E-value threshold) were visualized using the metagenomic software MEGAN [100]. Based on NCBI taxonomy, this software explores the taxonomic content of the samples with the option “import BLASTN”. The program uses several thresholds to generate sequence-taxon matches. The “min-score” filter, corresponding to a bit score cutoff value, was set at 35 for 16S rDNA amplicons as previously described [19], and at 200 for ITS2 amplicons to obtain an alignment with a minimum of 100 nucleotides. The “top-percent” filter used to select hits whose scores lay within a given percentage of the highest bit score, was set at 10 and at 5 for 16S rDNA and the ITS2 loci, respectively. The “min-support core” filter, used to set a threshold for the minimum number of sequences that must be assigned to a taxon, was set to 5. These stringent parameters should result in a “conservative” assignment of many sequences to internal branches (i.e. with less precision) of the taxonomic tree. Distribution of the sequences was schematically represented by Neighbor-Joining (NJ) tree diagrams (Figures S1, S2, S3, S4).

### Rarefaction curves and richness estimator

The quality of the sampling effort was assessed through the calculation of rarefaction curves, i.e. the number of operational taxonomic units (OTUs) with respect to the number of reads [101]. The prokaryote 16S rDNA and fungus ITS2 pyrosequences were sorted by decreasing length and clustered with 97% similarity using Uclust (http://www.drive5.com/usearch/) [102]. Rarefaction curves were calculated according to Uclust results using a perl script. The total richness of a community from a patient-sample was estimated using the Chao1 richness estimator [103]. This non-parametric estimator allows cross-sample comparison of species diversity. The Chao1 index was calculated from Uclust results using the formula: Chao1 = n+(n_1_*(n_1_−1))/(2*(n_2_+1)), where n is total number of OTUs, n_1_, the number of OTUs composed of one read, and n_2_, the number of OTUs composed of two reads. These diversity indices and richness estimators were then used to compare the relative complexities of communities and to estimate the completeness of sampling.

### Phylogenetic analysis

The phylogenetic trees inferred from 16S rDNA and ITS2 pyrosequences were used to compare biodiversity of specific taxa or within genera between samples for the same patient and/or between patients. The bacterial 16S rDNA sequences corresponding to the genera *Streptococcus*, *Haemophilus* and *Pseudomonas*, and the fungal ITS2 rDNA reads corresponding to those of *Aspergillus*, *Candida* and *Malassezia* were extracted from the pyrosequencing database using MEGAN, individually sorted by size, and clustered by homology (with a 97% identity threshold) using Uclust [102]. The longest read (>400 bp) from each cluster was selected as the representative sequence and submitted to a BLAST search [99] on the non-redundant nucleotide database (NCBI) to determine an approximate phylogenetic affiliation. The representative sequences and reference sequences were aligned using Muscle [102] as implemented in the SeaView4 program [104]. The resulting alignments were manually curated with the BioEdit software (http://www.mbio.ncsu.edu/bioedit/bioedit.html), and phylogenetic trees were constructed using both the NJ method from the SeaView4 package [104] and the Bayesian method implemented in MrBayes3 software (http://mrbayes.csit.fsu.edu/index.php) [105]. Since topologies of the phylogenetic trees generated by the two methods were similar, only the NJ trees are shown. The reliability of internal branches was assessed using the bootstrap method implemented in SeaView4 with 1000 replicates; only probabilities of more than 50% are shown at the tree nodes. Phylogenetic trees were edited using Dendroscope [106].

The pyrosequences were deposited in GenBank-SRA under the accession number SRA049426.2.

### Statistical analysis

Numerical variables were described as medians and interquartile ranges (Q1, Q3). To study the relationship between clinical data, taxon richness, and community composition of sputum samples, Spearman's correlation coefficient were calculated. P-values of less than 0.05 were considered as significant. All statistical analyses were performed using SAS software (SAS Institute, Cary, NC, USA; version 9.2).

## Supporting Information

Figure S1 **Taxonomic assignment of the 16S rDNA (A) and ITS2 (B) reads obtained from Patient 1.**
(TIF)

Figure S2 **Taxonomic assignment of the 16S rDNA (a) and ITS2 (b) reads obtained from Patient 2.**
(TIF)

Figure S3 **Taxonomic assignment of the 16S rDNA (a) and ITS2 (b) reads obtained from Patient 3.**
(TIF)

Figure S4 **Taxonomic assignment of the 16S rDNA (a) and ITS2 (b) reads obtained from Patient 4.** Footnotes for figures S1 to S4. Reads obtained from sputum samples of Patients 1–4 were analyzed using the software MEGAN, after BLASTN search against databases (see Material and Methods section). The MEGAN software plots on schematic trees represent the number of pyrosequence reads matching a particular taxonomical group. The tree displays all taxonomic groups identified from the assignment of reads obtained either with prokaryotic primers (Figures S1A–S4A), or fungus-designed primers (Figures S1B–S4B).
(TIF)

Figure S5 **NJ-phylogenetic tree of ITS2 sequences from the genus** ***Aspergillus*** **.**
(TIF)

Figure S6 **NJ-phylogenetic tree of 16S rRNA sequences from the genus** ***Pseudomonas*** **.**
(TIF)

Figure S7 **NJ-phylogenetic tree of 16S rRNA sequences from the genus** ***Streptococcus*** **.**
(TIF)

Figure S8 **NJ-phylogenetic tree of ITS2 sequences from** ***Candida albicans*** **.**
(TIF)

Figure S9 **NJ-phylogenetic tree of ITS2 sequences from the** ***Candida parapsilosis*** **complex.** Footnotes for figures S5 to S9. Neighbor-joining trees of the ITS2 or 16SrRNA sequences from the genus *Aspergillus* (Figure S5), *Pseudomonas* (Figure S6), and *Streptococcus* (Figure S7), and the species *C. albicans* (Figure S8) and the *C. parapsilosis* complex (Figure S9). The representative sequences corresponding to Patient 1 in blue, Patient 2 in green, Patient 3 in red and Patient 4 in yellow, while dark and light colour intensity were corresponding to the first and second sampling dates, respectively. Numbers in brackets indicate the number of reads composing each cluster. Clusters composed of reads that are at least 50% greater than the number of reads composing the most dominant cluster are in bold. Bootstrap values (threshold >50) are indicated at the nodes.
(TIF)
