# Supplementary figures and images for: The Airway Microbiota in Cystic Fibrosis: A Complex Fungal and Bacterial Community—Implications for Therapeutic Management
Source: PLoS One. 2012 Apr 27;7(4):e36313. doi: 10.1371/journal.pone.0036313 (PMC3338676; doi:10.1371/journal.pone.0036313)

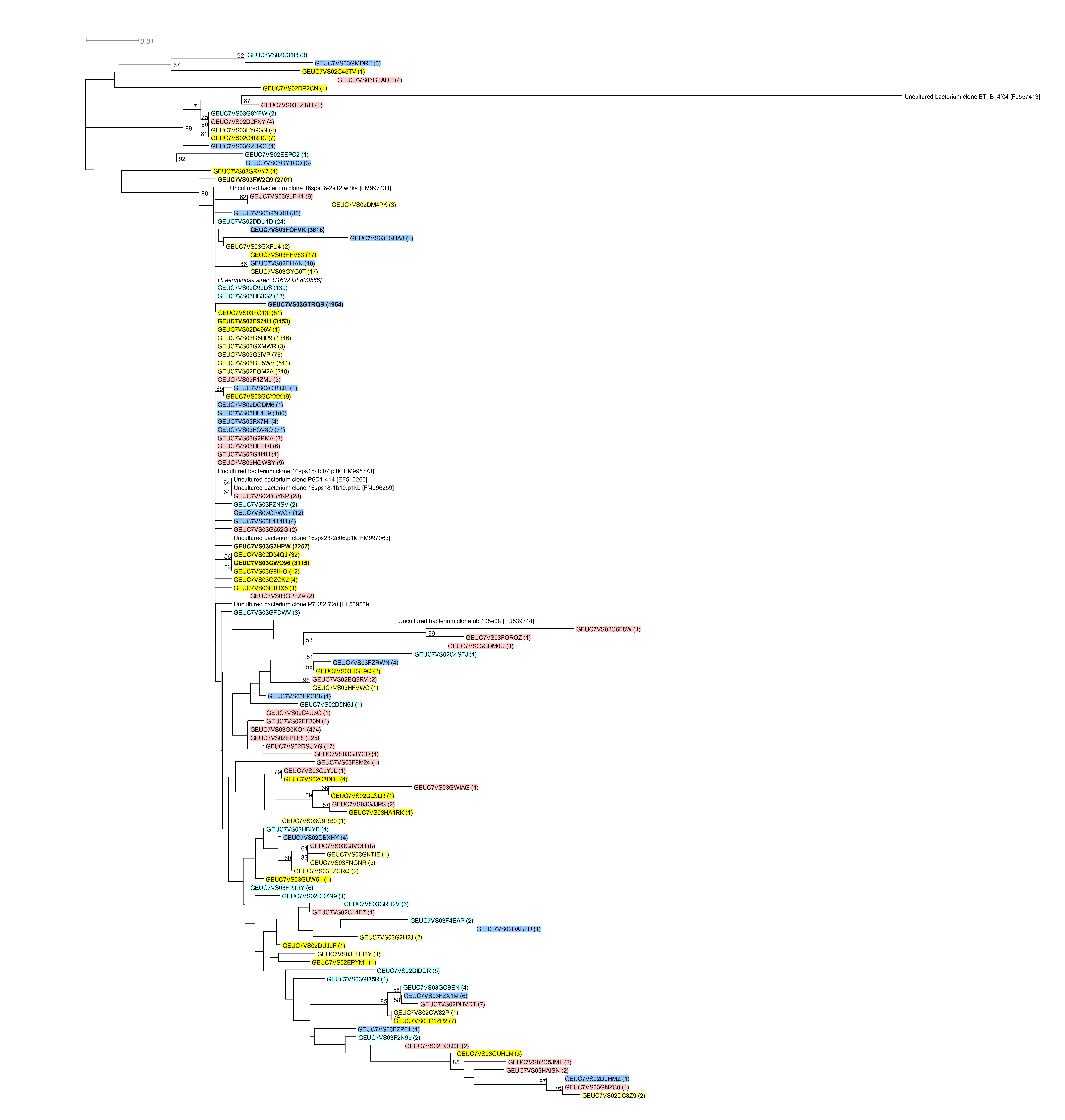

Supplement: Figure S6 — NJ-phylogenetic tree of 16S rRNA sequences from the genus Pseudomonas . (TIF) [file pone.0036313.s006.tif]

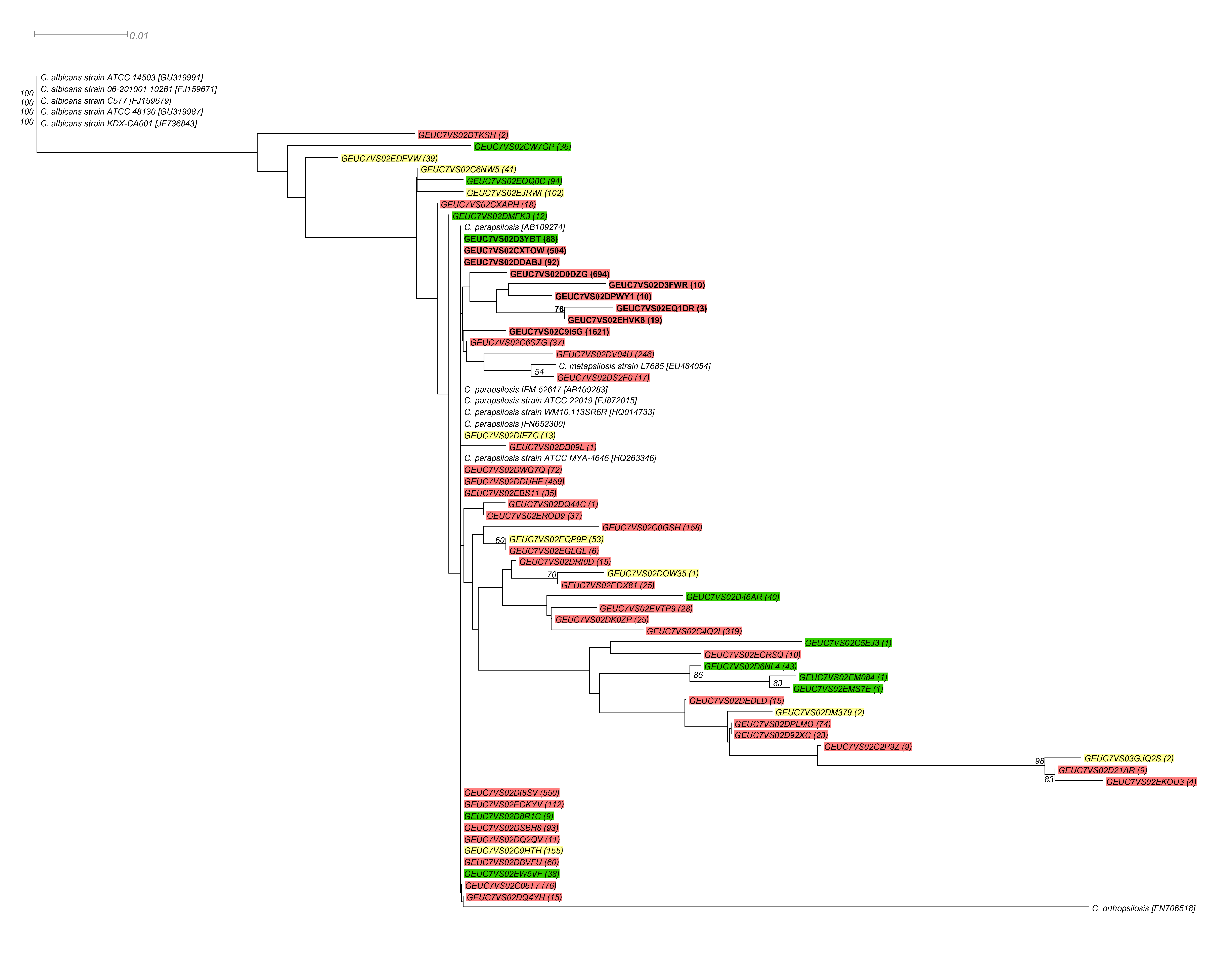

Supplement: Figure S9 — NJ-phylogenetic tree of ITS2 sequences from the Candida parapsilosis complex. Footnotes for figures S5 to S9. Neighbor-joining trees of the ITS2 or 16SrRNA sequences from the genus Aspergillus (Figure S5), Pseudomonas (Figure S6), and Streptococcus (Figure S7), and the species C. albicans (Figure S8) and the C. parapsilosis complex (Figure S9). The representative sequences corresponding to Patient 1 in blue, Patient 2 in green, Patient 3 in red and Patient 4 in yellow, while dark and light colour intensity were corresponding to the first and second sampling dates, respectively. Numbers in brackets indicate the number of reads composing each cluster. Clusters composed of reads that are at least 50% greater than the number of reads composing the most dominant cluster are in bold. Bootstrap values (threshold >50) are indicated at the nodes. (TIF) [file pone.0036313.s009.tif]
